# Overexpression of *OsGF14C* enhances salinity tolerance but reduces blast resistance in rice

**DOI:** 10.3389/fpls.2023.1098855

**Published:** 2023-02-10

**Authors:** Jingfang Dong, Xuezhong Li, Yamei Ma, Jianyuan Yang, Jiansong Chen, Wu Yang, Lian Zhou, Jian Wang, TiFeng Yang, Shaohong Zhang, Junliang Zhao, Qing Liu, Lingyan Zhou, Xiaoyuan Zhu, Bin Liu

**Affiliations:** ^1^ Rice Research Institute, Guangdong Academy of Agricultural Sciences, Guangzhou, China; ^2^ Guangdong Key Laboratory of New Technology in Rice Breeding, Guangzhou, China; ^3^ Guangdong Rice Engineering Laboratory, Guangzhou, China; ^4^ College of Agriculture and Biology, Zhongkai University of Engineering, Zhongkai, China; ^5^ Guangdong Key Laboratory of New Technology in Plant Protection, Plant Protection Research Institute, Guangdong Academy of Agricultural Sciences, Guangzhou, China

**Keywords:** OsGF14C, salinity, blast, Na+ Uptake, Lox2, rice

## Abstract

High-salinity and blast disease are two major stresses that cause dramatic yield loss in rice production. *GF14* (*14-3-3*) *genes* have been reported to play important role in biotic and abiotic stresses in plants. However, the roles of *OsGF14C* remain unknown. To understand the functions and regulatory mechanisms of *OsGF14C* in regulating salinity tolerance and blast resistance in rice, we have conducted *OsGF14C*-overexpressing transgenic experiments in the present study. Our results showed that overexpression of *OsGF14C* enhanced salinity tolerance but reduced blast resistance in rice. The enhanced salinity tolerance is related to the reduction of methylglyoxal and Na^+^ uptake instead of exclusion or compartmentation and the negative role of *OsGF14C* in blast resistance is associated with the suppression of *OsGF14E*, *OsGF14F* and *PR* genes. Our results together with the results from the previous studies suggest that the lipoxygenase gene *LOX2* which is regulated by *OsGF14C* may play roles in coordinating salinity tolerance and blast resistance in rice. The current study for the first time revealed the possible roles of *OsGF14C* in regulating salinity tolerance and blast resistance in rice, and laid down a foundation for further functional study and crosstalk regulation between salinity and blast resistance in rice.

## Introduction

Rice (*Oryza sativa* L.) is the staple food for over half of the world’s population. Therefore, rice production plays a crucial role in world food security. However, rice production is frequently subjected to biotic and abiotic stresses. High-salinity and blast disease are two major stresses that cause dramatic yield loss in rice production (Jacobs and Wang, 2021; Yin et al., 2021; Liu et al., 2022).

It has been reported that more than 955 million hectares of land (approximately 6.5% of the world’s total land area), 20% of the world’s cultivated land area and nearly 50% of the world’s irrigated land area are affected by salinity (Tufail et al., 2018), and this problem continues to worsen. In addition, the marginal lands such as coastal regions which are saline soils and unsuitable for crop cultivation need to be utilized for mitigating the issues of food demands.

Salinity tolerance in rice is a quantitative trait which is regulated by a host of genes in plants. It is imperative to understand the molecular mechanism of salinity tolerance in rice to minimize salinity related yield penalty. So far, a number of salinity tolerance genes involving in transcription regulation, signal transduction, ion transportation and metabolic homeostasis have been identified in plants (Apse et al., 1999; Shi et al., 2000; Singla-Pareek et al., 2003; Conti et al., 2008; Munns et al., 2012; Kesten et al., 2019; Ma et al., 2019; Zhao et al., 2019; Song et al., 2021; Ganapati et al., 2022). However, the molecular mechanisms underlying salinity tolerance are still not well understood. Particularly, the crosstalk between salinity and other stresses remain largely unknown (Gong et al., 2020; Zelm et al., 2020; Ganapati et al., 2022).

Rice blast disease caused by *Magnaporthe oryzae* is one of the most destructive rice diseases leading to severe yield losses in rice production worldwide (Yin et al., 2021). Rice blast resistance can be divided into qualitative resistance conferred by major genes (R) and quantitative (partial) resistance contributed by multiple genes (Kou and Wang, 2010; Fu et al., 2011). Despite the powerful and universally used of R genes, quantitative resistance is presumably non race specific and is generally considered to be broader spectrum and more durable in natural conditions (Kou and Wang, 2010).

The *GF14* (*14-3-3*) genes are ubiquitous in eukaryotic organisms. Plant GF14 proteins interact with a broad range of phosphorylated proteins and function in signaling, transcription activation or defense (Roberts, 2003), which endow *GF14* proteins different roles in plant development, metabolism, environmental stress responses and may establish a platform for crosstalk in diverse pathways (Oecking and Jaspert, 2009). Many *GF14* genes have been reported to be involved in biotic stresses in various crop plants. For example, the barley GF14 proteins have been demonstrated to interact with plasma membrane H^+^-ATPases in epidermal cells as they respond to infection by the powdery mildew fungus (Finni et al., 2002). The Arabidopsis GF14-λ confers resistance to the fungal pathogen *Golovonomyces* spp. through interaction with the RPW8.2 protein (Yang et al., 2009). The tomato GF14 protein 7 directly interacts with mitogen-activated protein kinase kinase kinase (MAPKKKa) to positively regulate immunity-associated programmed cell death (Oh et al., 2010). There are eight *GF14* members in rice (*GF14A* - *GF14H*) (Chen et al., 2006), among which, OsGF14B may interact with mitogen-induced MAP kinase 1 (BIMK1) which was induced by rice blast infection and participates in systemic acquired disease resistance (Cooper et al., 2003). *OsGF14E* was the first *GF14* gene confirmed to play a negative role in bacterial *Xoo* and fungal pathogen *Rhizoctonia solani* disease resistance (Manosalva et al., 2011). In previous study, we also found that *OsGF14E* positively regulates panicle blast resistance in rice (Liu et al., 2016b), while *OsGF14B* positively regulates panicle blast resistance but negatively regulates leaf blast resistance in rice (Liu et al., 2016a). In addition to the roles in biotic stresses, *GF14* genes have been also reported to function in abiotic stresses. Overexpression of the *Arabidopsis GF14λ* in cotton leads to a “stay-green” phenotype and improves stress resistance under moderate drought conditions (Yan et al., 2004). However, *GF14λ* and *GF14κ* were reported to negatively regulate salinity tolerance in *Arabidopsis* (Zhou et al., 2014). Co-expression of D-myo-inositol-3-phosphate synthase (IPS) and 14-3-3-like protein GF14 is responsible for the interaction between maize and *Rhizophagus intraradices* under drought stress, and potentially induces the synergistic actions of the symbiotic partners in enhancing plant drought resistance (Li et al., 2016). Ectopic expression of *TaGF14b* in tobacco led to longer root, better growth status, and higher relative water content, survival rate, photosynthetic rate, and water use efficiency in transgenic tobaccos compared to those in control plants under drought and salinity stresses (Zhang et al., 2018a). Studies also showed that the binding of GF14ω to Glutamate Receptor-Like Protein GLR3.7 inhibited primary root length under salinity stress in *Arabidopsis thaliana* (Wang et al., 2019). In addition to its function in blast, the *OsGF14B* was also found to regulate rice response to drought stress. The *OsGF14B*-overexpression lines exhibited enhanced sensitivity to drought and osmotic stress (Liu et al., 2019).

*OsGF14C* has been reported to be responsive to different biotic and abiotic stresses in rice (Chen et al., 2006). However, its actual functions in different stresses in rice have not been fully characterized yet. In the present study, we found that overexpression of *OsGF14C* showed opposite roles in response to salinity and blast stress in rice. The overexpression of *OsGF14C* positively regulates salinity tolerance by reducing the level of methylglyoxal and Na^+^ uptake instead of exclusion or compartmentation, while negatively regulates blast resistance by inhibiting the expression of *GF14E*, *GF14F* and *PR* genes. Furthermore, our results and the previous studies together implied that the lipoxygenase gene *LOX2* which is regulated by *OsGF14C* may play roles in both salinity tolerance and blast resistance in rice. Thus, the present study for the first time revealed the possible roles of *OsGF14C* in regulating salinity tolerance and blast resistance in rice, and provided insights into the crosstalk regulation between salt and blast resistance in rice.

## Materials and methods

### Vector construction and genetic transformation

For *OsGF14C* overexpression vector construction, the coding region sequence of *OsGF14C* was amplified from the blast-resistant line BC10 using the primers in **Supplemental Table 1**. The resulting product (843bp) was cloned into pEASY-T1 (TransGen) vector and verified by sequencing. The entry clone for overexpressing plants was then inserted into PHQSN (modified from pCAMBIA1390) which harbors a CaMV*35S* promoter. The positive plasmid was electroporated into *Agrobacterium tumefaciens* EHA105 and then introduced into calli of the cultivar *Lijiangxintuanheigu* (LTH) *via* Agrobacterium-mediated genetic transformation.

### RNA extraction and quantitative real-time PCR (qRT-PCR)

Total RNA was extracted with Trizol reagent (Invitrogen) and purified with NucleoSpin RNA Clean-up (MACHEREYNAGEL) according to the manufacturers’ instructions. RNA quality and quantity were assessed by formaldehyde denaturing agarose gel electrophoresis and spectrophotometry (Nanodrop-1000), respectively. The purified total RNA was reverse-transcribed using the Primescript™ RT reagent kit (Takara) to generate cDNA and real-time PCR was carried out using SYBR ExTaq™ (Takara). *Actin* gene was chosen as a reference gene. Gene expression was quantified by the comparative CT method. Experiments were performed in triplicate, and the results were presented by their means ± standard derivation (SD). Gene-specific primers used were listed in **Supplemental Table 1**.

### Salinity treatment and evaluation of salinity tolerance

We got *OsGF14C* over expression plants by vector construction and rice transformation. The relative expression level of *OsGF14C* in every line was measured by quantitative real-time PCR. The seeds of T_0_, T_1_, and T_2_ segregating progeny were used for salinity treatment. After incubation at 49°C for 96 h to break dormancy, seeds were soaked in water for 36 h and then incubated at 30°C for 48 h for pre-germination. The seeds that germinated uniformly were sown in cultural boxes with kimura nutrient mixture and grown in the tissue culture incubator for ~10 d. The kimura nutrient mixture was changed into new kimura nutrient mixture containing 120 mmol/L Nacl. After 7 d treatment, the culture solution was change back to normal kimura nutrient mixture without Nacl. After ~10 d recovery, the survival rate was calculated. The treatment was repeated three times.

### Measurement of electrolyte leakage

Electrolyte leakage was measured using the method reported in our previous study (Dong et al., 2019). Briefly, at 0 h, 2 d, 3 d, 4 d before and after Nacl treatment, 0.1 g of leaf material was collected and washed several times in ultrapure water and then immersed in 20 mL ultrapure water for 24 h. The electrical conductivity of the resulting solution was measured and defined as R1. This solution was placed in boiling water for 20-30 min. After cooling, the electrical conductivity was measured and defined as R2. Relative electrolyte leakage (%) = (R1/R2)×100%. The experiment were repeated three times.

### Blast treatment and evaluation of disease resistance

We got *OsGF14C* over expression plants by vector construction and rice transformation. Wild-type LTH plants and 15 lines of T_1_ segregating progeny germinated from T_0_ transgenic seeds were grown in soil in greenhouse. The relative *OsGF14C* transcription level was measured by quantitative real-time PCR. *M. oryzae* isolate GD08-T13 inoculum was used for blast resistance evaluation as described in our previous study (Dong et al., 2021). Briefly, when the plants grow to tillering stage, the leaves of the plants were inoculated with GD08-T13 using the punch method. The inoculated plants were sprayed with water for 2-3 min every 2 h to maintain the humidity. After 10 days inoculation, the lesion size of each leaf was measured. The correlation between the disease area and relative *OsGF14C* transcription was analysed in excel. The treatment was repeated three times.

### Sub-cellular localization analysis

We amplified the protein coding region of *OsGF14C* from BC10 using the primers in **Supplemental Table 1** and cloned it into the pUC18 vector with the coding sequence for enhanced green fluorescent protein (eGFP) under the control of the Cauliflower mosaic virus (CaMV) 35S promoter provided by Yang et al. (2014). Dehulled rice seeds of LTH were surface-sterilized using 1.5% sodium hypochlorite for 40 min, then germinated and cultured on half-strength Murashige and Skoog medium in the tissue culture room for ~10 d. Seedlings were grown hydroponically under natural light for 3 d. The protoplast isolation and transformation were performed following the method of Yang et al. (2014). After 24 h incubation at 28°C without light, the rice protoplasts were observed and photographed under a laser confocal microscopy (Zeiss LSM710, Germany).

### Measurement of methylglyoxal content

The samples were ground in liquid nitrogen to powder. 1 g of sample was dissolved in 5 mL of water, and placed in an ice water bath for ultrasonic extraction for 1 h. After centrifugation at 4°C, 12000 rpm for 5 min, 1 mL supernatant extract was mixed with 1 mL aqueous anthalenediamine solution (6 g/L), derivatization reaction was performed for 8 h at room temperature and dark conditions, filtered at 0.22 um, and then conducted chromatograph analysis. Chromatographic conditions are as follows: chromatographic column: Kromasil; Anti-phase chromatography column (150 mm × 4.6 mm, 5 um); Mobile phase: 0.1%(V/V) acid aqueous solution (A) and methyl alcohol (B); gradient elution program: 0~5 min: B= 30%; 5~10 min: B up from 30% gradient to 90%; 10~15 min: B= 90%; 15~16 min: B gradually decreased from 90% to 30%; 16~20 min: B= 30%; strength: 1.0 ml/min. Detection wavelength: 318nm; Column temperature: 30°C; Sample volume: 10uL; Retention time: 10.3min. Methylglyoxal content= Detection concentration*Sample extraction volume (dilute volume)/the weight of sample.

## Results

### *OsGF14C* is induced by salinity stress but repressed by blast infection

**Figure 1:**
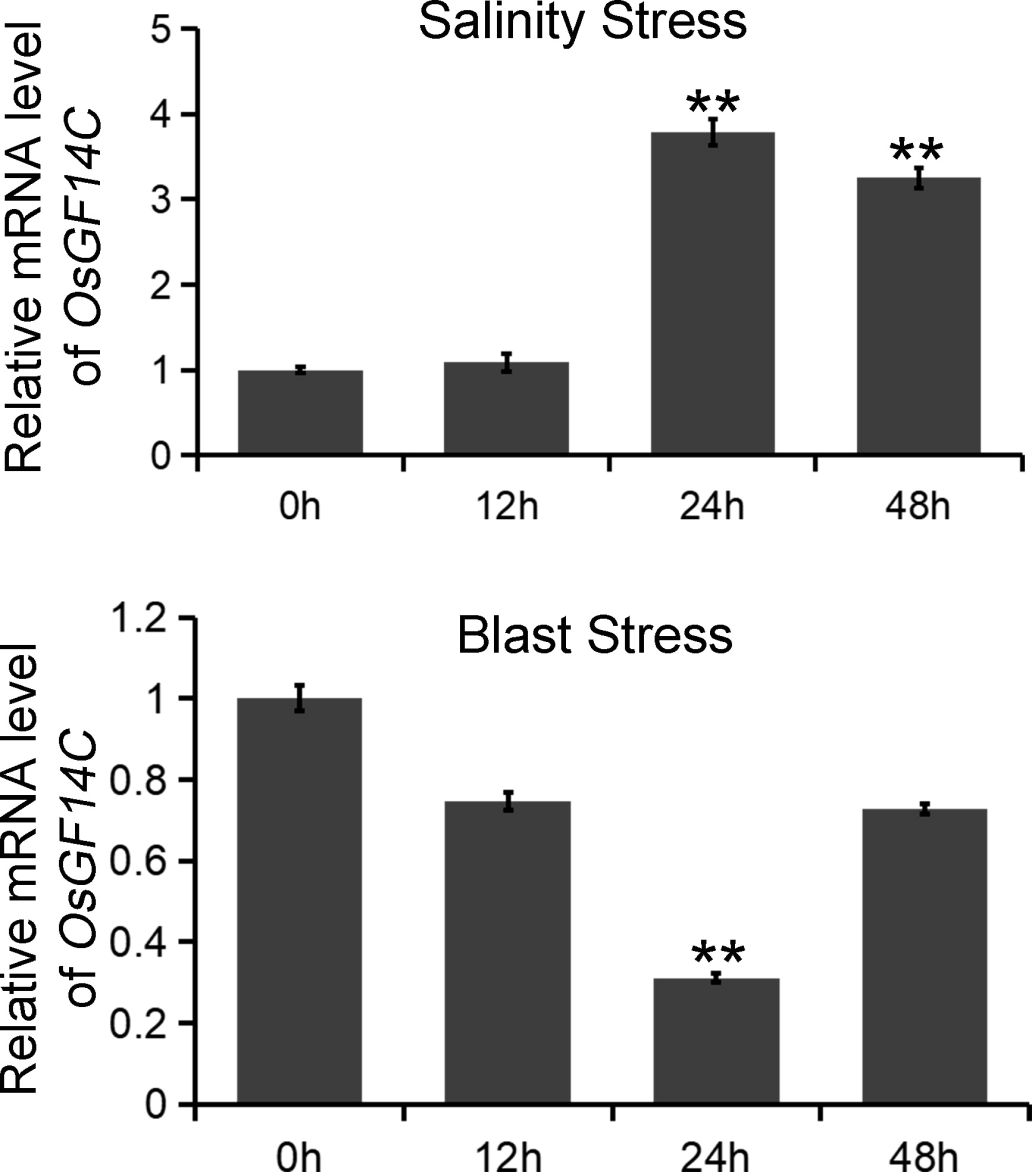
The expression patterns of *OsGF14C* in responses to salinity stress and blast stress, respectively. The expression of *OsGF14C* during salinity stress and blast stress as assessed by quantitative real-time PCR at 0 h before treatment and 12 h, 24 h, 48 h after treatment. Error bars indicate the standard deviation (SD) from three biological replicates and ** indicates a statistically significant difference compared with 0 h treatment (*t* test, *p* < 0.01).

To investigate the biological functions of *OsGF14C* in salinity and blast resistance in rice, we first analyzed the expression patterns of *OsGF14C* under salinity stress and blast infection. The experiments were conducted at 0h, 12h, 24h and 48h after stress treatments. The results indicated that the expression levels of *OsGF14C* significantly increased at 24h and 48h after salinity treatment, while significantly decreased at 24h after blast inoculation (**Figure 1**).

### Overexpression of *OsGF14C* enhances tolerance to salinity stress but reduces resistance to blast in rice

To confirm the functions of *OsGF14C* in salinity and blast resistance in rice, we produced the *OsGF14C* overexpressing plants (*OsGF14C*-OX) using the variety Lijiangxintuanheigu (LTH) as material, which is susceptible to blast. Fifteen independent transgenic lines were generated and the OX plants showed no differences in morphological phenotypes compared to wild type plants.

**Figure 2:**
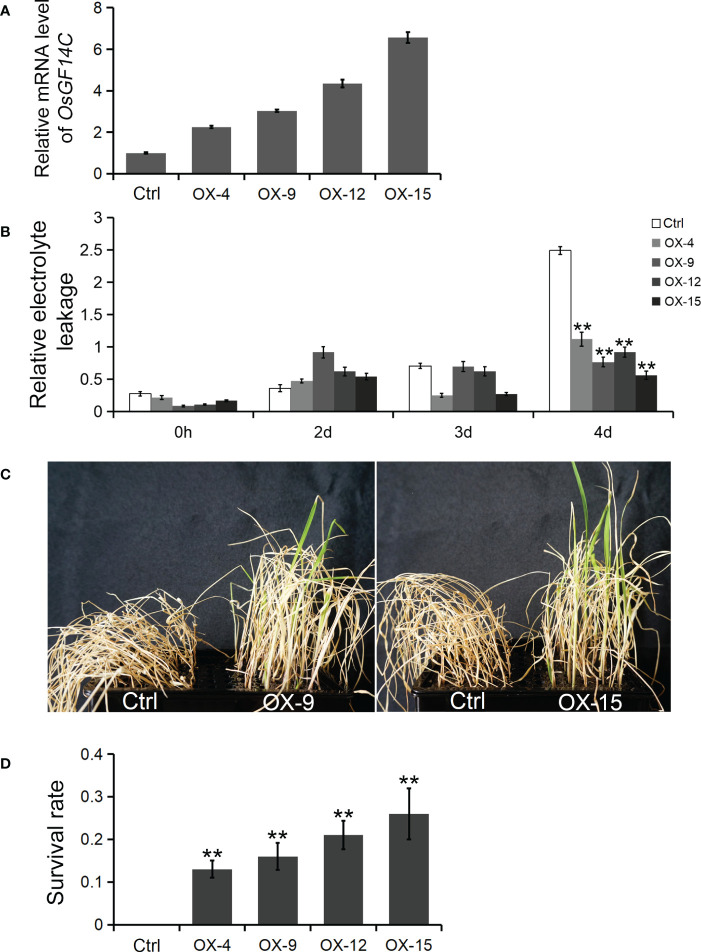
The role of *OsGF14C* in resistance to salinity stress in rice. **(A)**, the relative *OsGF14C* transcription level of four independent homozygous lines (OX-4, OX-9, OX-12 and OX-15) used in salinity tolerance evaluation experiment; **(B)**, the relative electrolyte leakages of Ctrl, OX-9, and OX-15 on the forth day after salinity treatment; **(C)**, the growth state of Ctrl, OX-9, and OX-15 after seven days of salinity treatment and ten days of recovery; **(D)**, the survival rate of Ctrl, OX-4, OX-9, OX-12 and OX-15 after seven days of salinity treatment and ten days of recovery. Error bars indicate the standard deviation (SD) from three biological replicates and ** indicates a statistically significant difference compared with 0 h treatment (*t* test, *p* < 0.01).

**Figure 3:**
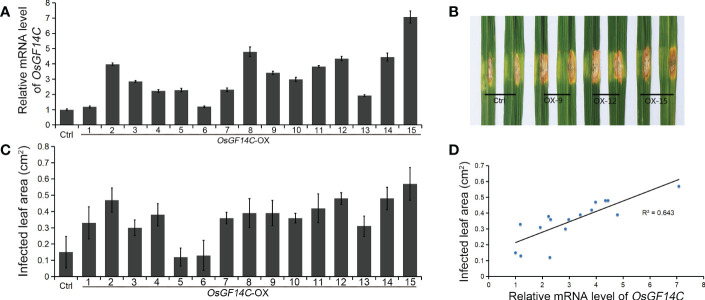
The role of *OsGF14C* in resistance to blast stress in rice. **(A)**, the relative *OsGF14C* transcription of independent 15 *OsGF14C*-OX lines; **(B)**, the infected lesions areas of Ctrl and *OsGF14C*-OX lines after inoculation using punch method; **(C)**, the infected lesions areas on leaves of the 15 *OsGF14C*-OX lines; **(D)**, the correlation between **(A, C)**.

Four independent homozygous lines (OX-4, OX-9, OX-12 and OX-15) with different high transcription levels of *OsGF14C* were selected for evaluation of salinity tolerance (**Figure 2A**). The results showed that the relative electrolyte leakages, which is an indicator of membrane injury under salinity stress, of all the *OsGF14C*-OX lines were significantly lower than the wild-type plants on the fourth day after high salinity stress treatment (**Figure 2B**). After salinity stress treatment for seven days and then recovery for ten days, the survival rates of the *OsGF14C*-OX lines were significantly higher than their wild-type plants (**Figures 2C, D**). We also measured the transcription level of *OsGF14C* in the fifteen OX lines and conducted blast resistance evaluation using *M. oryzae* isolate GD08-T13 by measuring the infected lesion areas (**Figures 3A–C**). Correlation analysis demonstrated a positive correlation between the expression level of *OsGF14C* and infected leaf area (R^2 =^ 0.643; **Figure 3D**), suggesting that increased *OsGF14C* expression may reduce the blast resistance in rice.

### *OsGF14C* is ubiquitously expressed in different tissues and localized in cytoplasm

**Figure 4:**
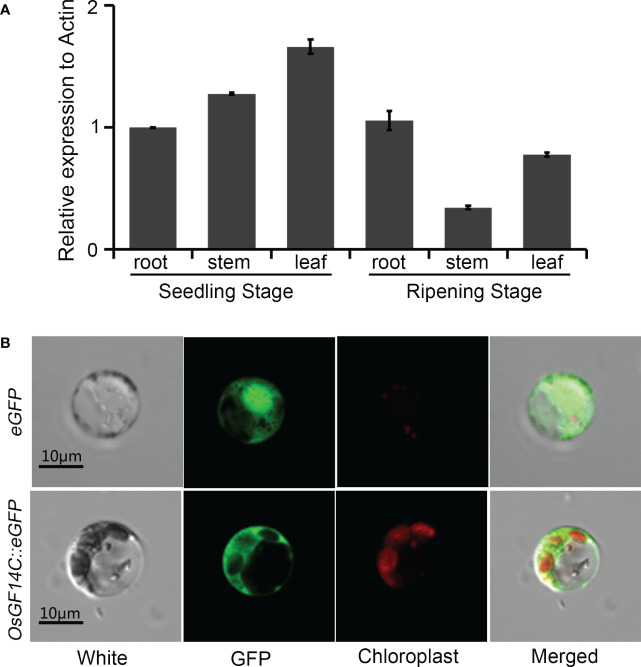
The temporal and spatial expression patterns of *OsGF14C* and its subcellular localizations. **(A)** Expression analysis of *OsGF14C* in different rice tissues by quantitative real-time PCR. Error bars indicate the standard deviation (SD) from three biological replicates; **(B)** Sub-cellular localization of *OsGF14C* in rice protoplasts. White indicates the protoplast under visible light, GFP indicates the eGFP fluorescence, Chloroplast indicates the chloroplast fluorescence, Merge indicates the overlapping situation of White, GFP, and Chloroplast.

In order to gain further insights into the function of *OsGF14C* in rice response to stresses, the temporal and spatial expression patterns of *OsGF14C* in root, stem and leaf at different stages were analyzed. As shown in **Figure 4A**, the expression of *OsGF14C* can be detected in all rice tissues examined in the present study and relative higher expression level was observed in leaf at seedling stage.

To investigate the sub-cellular localization of *OsGF14C*, the coding domain sequence of *OsGF14C* was fused with the green fluorescence protein (*eGFP*) under the control of the cauliflower mosaic virus 35S promoter. The *OsGF14C::eGFP* fusion vector and the control vector (empty *eGFP* vector) were transiently expressed in rice protoplasts. The laser confocal microscope showed that the green fluorescent signal in the protoplasts expressing *eGFP* control vector was distributed both in the nucleus and the cytoplasm, whereas the green fluorescent signal was distributed in the cytoplasm of the rice protoplasts transfected with the *OsGF14C::eGFP* vector (**Figure 4B**). The results demonstrated OsGF14C was localized in cytoplasm.

### *OsGF14C* modulate the expression of defense-related genes and Na^+^ transport-related genes

According to the previous studies, the defense-related genes play important roles in blast resistance in rice, and the accumulation of toxic ions, such as Na^+^ is the main reason for ion toxicity in rice under salinity stresses (Yan et al., 2022). To elucidate the regulatory mechanisms underlying *OsGF14C* in response to salinity and blast stresses in rice, we detected the expression changes of defense-related genes and Na^+^ transport-related genes in transgenic and wild type plants.

**Figure 5:**
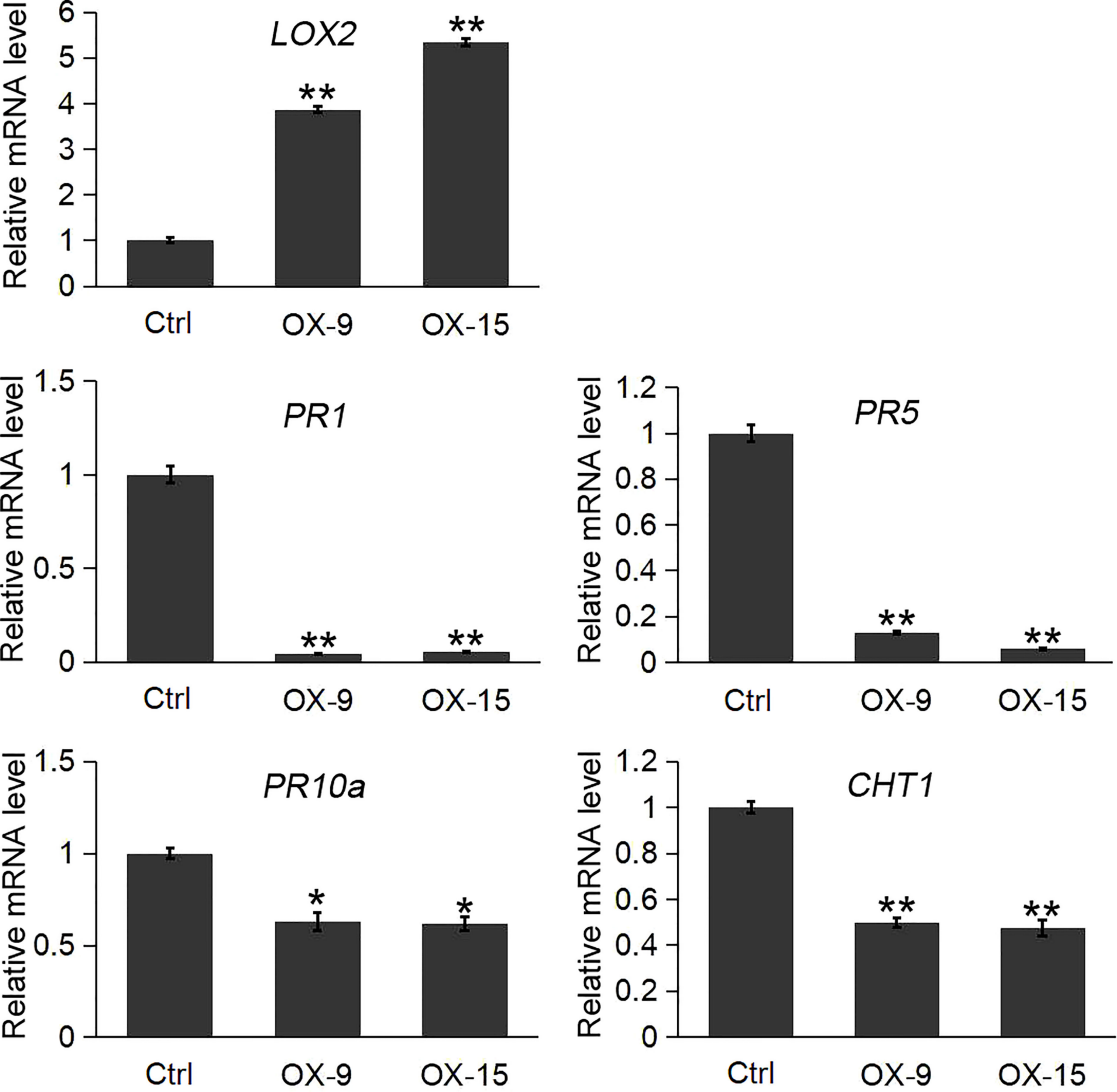
Expression analysis of *LOX2* and pathogenesis-related (*PR*) genes in the *OsGF14C*-OX transgenic plants and control plants (Ctrl) by quantitative real-time PCR. Error bars indicate the SD from three biological replicates and asterisks indicate statistically significant differences compared to the control plants (*t* test, **P < 0.01; *P < 0.05).

The results indicated that the expression of the lipoxygenase gene *LOX2* was significantly induced in *OsGF14C*-OX lines compared to the wild type plants. In contrast, the expression levels of three pathogenesis-related genes (*PR1*, *PR5*, *PR10a*) and a chitinase gene (*CHT1*, also named *PR-3* chitinase 1), were significantly lower in *OsGF14C*-OX lines than that in wild type plants (**Figure 5**).

**Figure 6:**
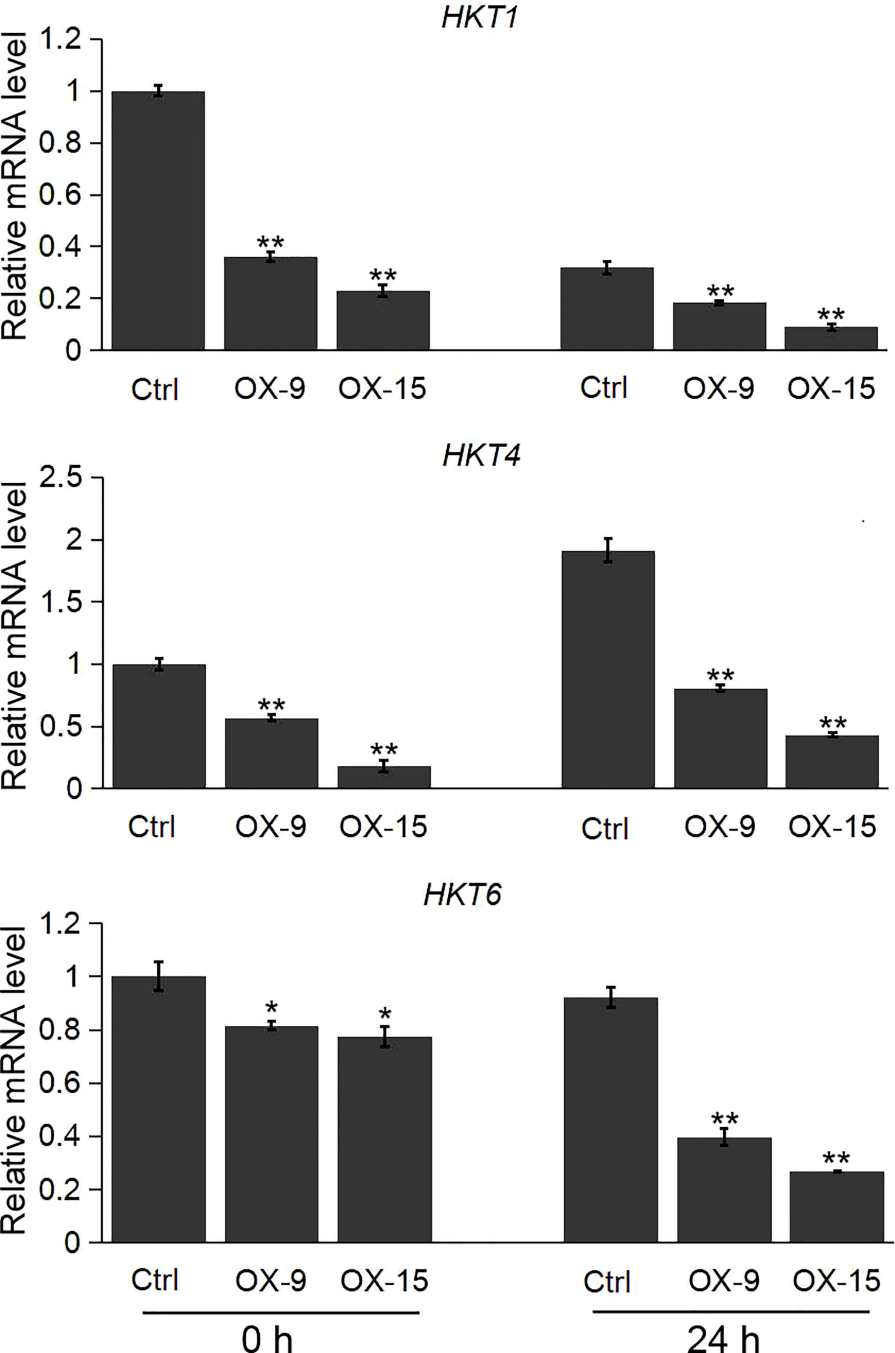
Expression analysis of *HKT* genes in the *OsGF14C*-OX transgenic plants and control plants (Ctrl) by quantitative real-time PCR. Error bars indicate the SD from three biological replicates and asterisks indicate statistically significant differences compared to the control plants (*t* test, **P < 0.01; *P < 0.05).

We also evaluated the expression pattern of the *HKT*, *NHX* and *SOS1* genes, which are the important player in regulating the ion homeostasis in rice under salinity stress. Our results showed that the expression levels of the three *HKT* genes (*HKT1*, *HKT4*, *HKT6*) were significantly lower in *OsGF14C*-OX lines than that in wild type plants both before and after salinity stress treatment (**Figure 6**). However, there was no significant difference in the expression levels of *NHX1*, *NHX5*, and *SOS1* between the *OsGF14C*-OX lines and the wild type plants (**Supplemental Figure 1**).

### The expression levels of the client proteins were differently regulated by *OsGF14C*

**Figure 7:**
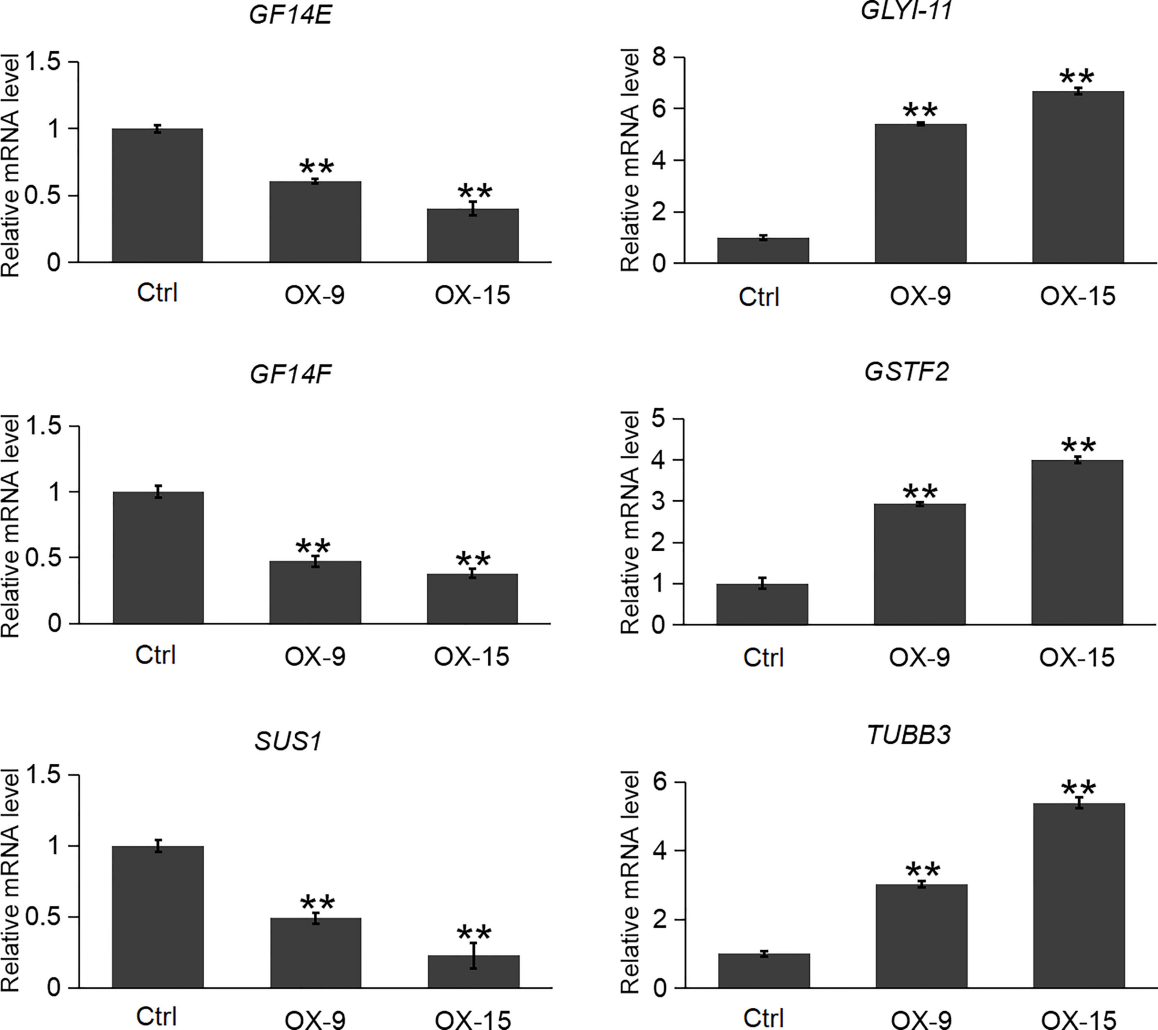
Expression analysis of several clients genes in the *OsGF14C*-OX transgenic plants and control plants (Ctrl) by quantitative real-time PCR. Error bars indicate the SD from three biological replicates and asterisks indicate statistically significant differences compared to the control plants (*t* test, **P < 0.01).

Plant GF14 proteins usually interact with a broad range of proteins to exert their functions. In the previous study, Zhang et al. (2017) reported 29 (exclude OsGF14C itself) client proteins of OsGF14C in rice root by affinity chromatography. Among the 29 client protein genes, 22 of them were found to be expressed in our *OsGF14C*-OX RNA-seq results. Among these 22 genes, only 6 of them had significant expression changes in *OsGF14C*-OX plants compared to wild-type plants (**Table S2**). In order to confirm these results, we further evaluated the expression levels of these gene by qRT-PCR. The results demonstrated that the expression levels of two other *OsGF14* genes (*GF14E* and *GF14F*) and a sucrose synthase gene *SUS1* were significantly lower in *OsGF14C*-OX lines than that in the wild type plants. In contrast, the expression levels of two self-defense mechanism related genes (*GLYI-11* and *GSTF2*), as well as a cell structure and growth-related gene *TUBC3* were significantly higher in *OsGF14C*-OX lines than that in the wild type plants (**Figure 7**).

## Disscussion

### Overexpression of *OsGF14C* positively regulates salinity tolerance while negatively regulates blast resistance in rice

It has been reported that *GF* (*14-3-3*) played very important roles in plant growth and development, as well as in response to various stresses. There are eight *GF* genes in rice, named *GF14A*-*GF14H*. The other studies and our previous studies have confirmed that *GF14B* and *GF14E* functioned in regulating disease resistance and drought tolerance in rice (Manosalva et al., 2011; Liu et al., 2016a; Liu et al., 2016b). Although it was reported that *OsGF14C* responded differently in various biotic and abiotic stresses in rice (Chen et al., 2006), the molecular functions of *OsGF14C* involved in biotic and abiotic stresses resistances in rice remain to be elucidated. In present study, the sub-cellular localization of OsGF14C in rice protoplasts revealed that OsGF14C accumulated only in the cytoplasm, which is consistent with the observations in rice callus and onion epidermal cells (**Figure 4B**). Differently, GF14B, GF14E and GF14F were localized in both nuclei and cytoplasm (Chen et al., 2006). Furthermore, the temporal and spatial expression analysis indicated that *OsGF14C* exhibited higher expression levels in all the tissues examined in the present study at the seedling stage compared to those at the ripening stage (**Figure 4A**). The different sub-cellular localization, temporal and spatial expression patterns imply that *OsGF14C* might have different functions in rice. Our transgenic experiments further confirmed this speculation.

Our results showed that the expression of *OsGF14C* was induced by salinity stress while suppressed by blast infection (**Figure 1**). The relative electrolyte leakages, which is an indicator of salinity resistance, of the *OsGF14C*-OX lines were significantly lower than that of the wild-type plants after salinity stress treatment (**Figure 2B**). In consistence with these results, after salinity treatment for seven days and then recovery for ten days, the survival rates of the *OsGF14C*-OX lines were significantly higher than that of their wild type plants (**Figures 2C, D**). On the contrary, for the blast disease resistance, the infected lesion areas of *OsGF14C*-OX lines were larger than the wild type plants and the expression levels of *OsGF14C* was positively correlated with infected leaf areas in *OsGF14C*-OX lines (**Figure 3**), indicating negative effect of *OsGF14C* in regulating blast resistance in rice. Taken together, our results suggest that overexpression of *OsGF14C* can improve salinity tolerance but reduce blast resistance in rice.

### The negative role of *OsGF14C* overexpression in blast resistance is associated with the suppression of *GF14E*, *GF14F* and *PR* genes

It is well known that *PR* genes play important roles in plant defense responses (Kaur et al., 2017). Our previous study and other studies also suggest that the *PR* genes are involved in blast resistance in rice (Tezuka et al., 2019; Dong et al., 2021). *CHT1*, which is also named as pathogenesis related (*PR*)-3 chitinase 1, is another typical defense response gene. Previous studies showed that *CHT1* played important roles in disease resistances, such as sheath blight disease and blast disease in rice (Hao et al., 2012). To investigate whether *OsGF14C* mediated blast resistance is associated with *PR* genes, we analyzed the expression of the *PR* genes in the present study. The results showed that the expression levels of *PR1*, *PR5*, *PR10a* and *CHT1* in *OsGF14C* overexpressing lines were all significantly lower than that in wild type plants, suggesting that *OsGF14C* negatively regulates blast resistance in rice by suppression of *PR* genes.

In plants, GF proteins are known to be involved in regulating a large number of biological processes *via* their interaction with numerous client proteins in a phosphorylation-dependent manner. Recently, 87 client proteins were identified through affinity chromatography assay using four GF proteins in rice, including the GF14C (Zhang et al., 2017). Twenty-nine (exclude OsGF14C itself) out of the 87 client proteins can interact with OsGF14C. Among these 29 client proteins, the expression levels of *OsGF14E* and *OsGF14F*, were significantly lower in *OsGF14C*-OX plants than that in wild type plants. In our previous study, we have functionally confirmed that *OsGF14E* and *OsGF14F* could positively regulate blast resistance in rice (Liu et al., 2016b; Ma et al., 2022). These results suggest that *OsGF14C* might negatively regulate blast resistance by suppressing the expression of *OsGF14E* and *OsGF14F*. However, further studies are needed to elucidate whether *OsGF14C* regulates blast resistance by an indirect way or by both indirect and direct ways.

### *OsGF14C* positively regulates salinity tolerance by reducing the level of methylglyoxal and Na^+^ uptake

**Figure 8:**
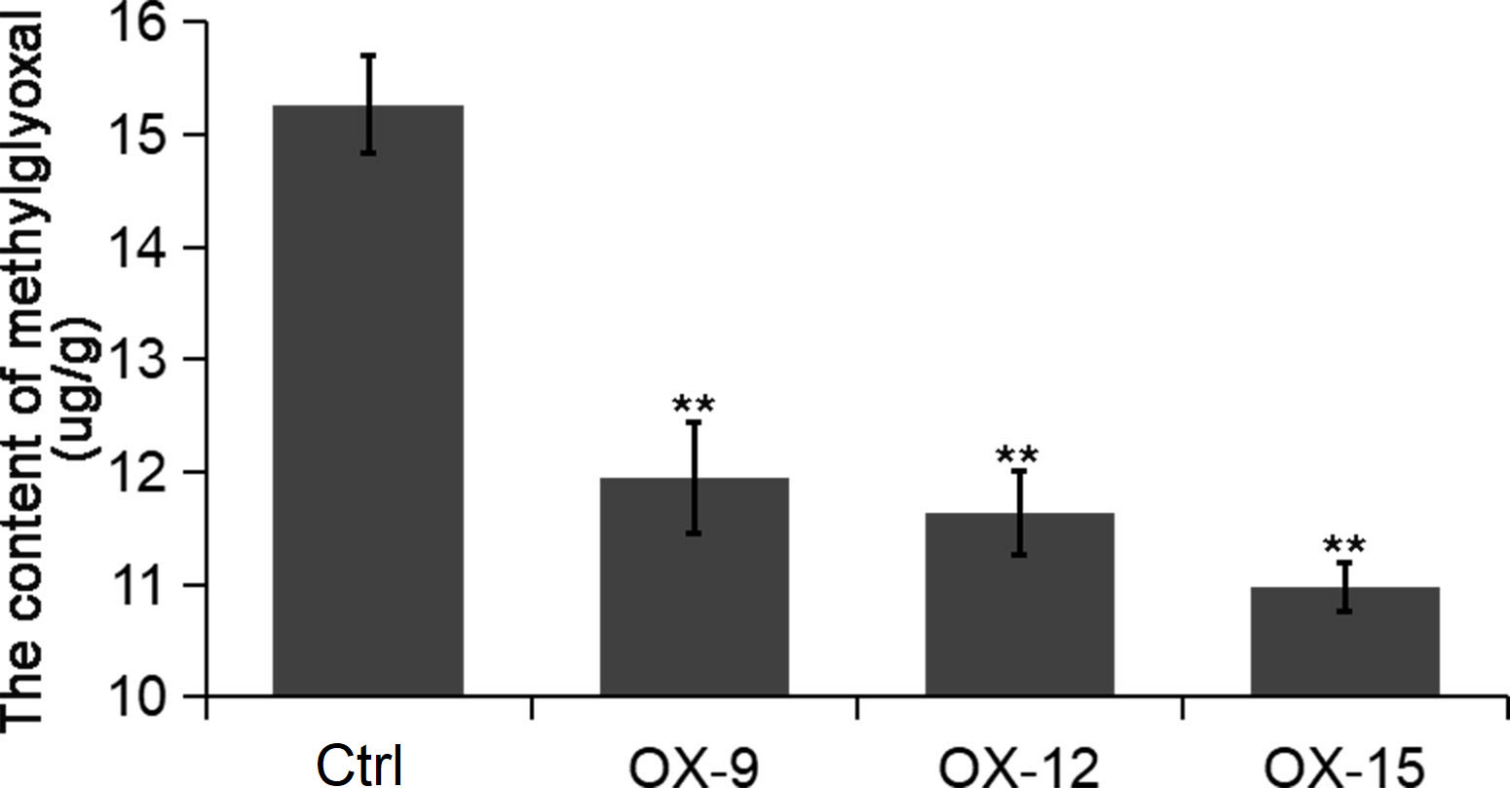
The content of methylglyoxal in the *OsGF14C*-OX transgenic plants and control plants (Ctrl). Error bars indicate the SD from three biological replicates and asterisks indicate statistically significant differences compared to the control plants (*t* test, **P < 0.01).

GFP (Glyoxalase family protein, also named GLY or GLO) and GSTF2 (Glutathione S-transferase) are another two client proteins of OsGF14C reported by Zhang et al. (2017). GLYI catalyzes methylglyoxal (MG) metabolism in the presence of GSH and produces an intermediate compound *S*-lactoylglutathione. According to previous studies, MG is a potent cytotoxic compound produced from glycolysis. The rate of glycolysis increases under stresses, and lead to MG accumulation in plants. MG accumulation is toxic to cells as it inhibits cell proliferation and results in a number of adverse effects, such as increasing the degradation of proteins and inactivating the antioxidant defense system (Hossain et al., 2009). Previous studies showed that overexpression of *GLY I+II* together in tobacco led to enhancement of salinity and zinc tolerance (Singla-Pareek et al., 2003; Singla-Pareek et al., 2006). Heterologous expression of *OsGLYI-11.2* in *Escherichia coli* and tobacco also resulted in improved adaptation to various abiotic stresses caused by increased scavenging of MG, lowering the Na^(+)^/K^(+)^ ratio and maintenance of reduced glutathione levels (Mustafiz et al., 2014). In the present study, the expression levels of *GLYI-11* and *GSTF2*, and the contents of MG were significantly lower in *OsGF14C*-OX lines than that in wild type plants (**Figures 7**, **8**). Taken together, these results suggest that the positive role of *OsGF14C* in regulating salinity tolerance might be partially attributed to its interactions with *GLYI-11* and *GSTF2*, and leading to the reduction of MG.

Plants have developed intricate mechanisms for Na^+^ uptake, export and compartmentation to reduce the accumulation of Na^+^ (Wu, 2018). So far, the salt-overly-sensitive (SOS) signaling pathway has been considered an importance pathway in salt resistance (Gong et al., 2020; Zelm et al., 2020). The *SOS1* encodes a plasma membrane Na^+^/H^+^ antiporter, which uses the H^+^ gradient to drive Na^+^ efflux and thus reducing cytosolic Na^+^ concentration (Shi et al., 2003). However, in the present study, there was no significant difference in the expression levels of *SOS1* between *OsGF14C*-OX plants and wild type plants, either before or after salinity treatment (**Supplemental Figure 1**). In addition, the central vacuole is an ideal location for Na^+^ sequestration to reduce the Na^+^ accumulation in the cytoplasm. NHX-type transporters play important roles in Na^+^ translocation from cytoplasm to vacuole through a Na^+^/H^+^ exchanger (Blumwald and Poole, 1985; Gong et al., 2020). Nevertheless, our results showed that the expression levels of *NHX1* and *NHX5* in *OsGF14C*-OX lines were similar to that in wild type plants, both with and without salinity treatment (**Supplemental Figure 1**). In contrast, the expression levels of HKT-type transporters including *HKT1*, *HKT4*, and *HKT6*, which mediate the translocation of Na^+^ from extracellular to cytoplasm in *OsGF14C*-OX plants were significantly lower than that in wild type plants, both before and after salinity treatment (**Figure 6**). Taken together, these results suggest that overexpression of *OsGF14C* may positively regulate salinity tolerance by lowering the expression of HKT-type transporter genes and inhibiting the Na^+^ uptake instead of exclusion or compartmentation.

### *LOX2* may play important roles in coordinating salinity tolerance and blast resistance in rice

In natural environment, plants are often subjected to both abiotic (such as drought, salt, cold) and biotic (necrotrophic and biotrophic pathogens) stresses simultaneously, which likely demands cross-talk between stress-response pathways to minimize the fitness costs. It is essential to find out the genes which could play roles in different stresses resistance and understand how the various stress response pathways interact with one another. In the present study, overexpression of *OsGF14C* enhanced the resistance to salinity stress but reduced the resistance to blast in rice. The opposite roles of *OsGF14C* overexpression played in salinity stress and blast disease are consistent with the previous findings that plants exposed to abiotic stresses such as high salinity and drought often display reduced immune activity (Bostock et al., 2014). It is understandable for this conflict as stress responses are costly, plants likely coordinate these responses to minimize fitness costs.

According to previous studies, LOXs, the key enzymes of JA synthesis, have been reported to involve in various physiological processes, including responses to biotic and abiotic stresses (Mostofa et al., 2015). Chu et al. (2019) showed that the *Arabidopsis* plants inoculated with *Pseudomonas* PS01 could survive under 225 mM NaCl, while the non-inoculated plants were dead above 200 mM NaCl. The gene expression analysis of the genes related to stress resistance indicated that only *LOX2* was up-regulated in the inoculated plants in comparison to the non-inoculated controls (Chu et al., 2019). Furthermore, Zhang et al. (2018b) found that *LOX2* and *LOX5* were specifically suppressed by Guy11 infection, which are the key JA synthesis genes hijacked by *M. oryzae* strain Guy11 to subvert host immunity and facilitate pathogenicity. They also proposed that induced expression of *OsLOX2/5* may improve resistance to the blast disease in rice (Zhang et al., 2018b). In the present study, our results showed that the expression levels of *LOX2* were significantly higher in the *OsGF14C*-OX plants than that in wild type plants. Given that *LOX* genes play important roles in regulating both biotic and abiotic stresses in plants, the increased expressions of *LOX2* in *OsGF14C*-OX plants implied *LOX2* may partially lead to the differences responses of transgenetic plants to salinity and blast resistance. However, despite that *OsGF14C*-OX plants showed increased expression of *LOX2*, the transgenetic plants were more sensitive to rice blast disease than their wild type plants, which seems to be conflict with previous studies. But we also detected reduced expression of some *PR* genes in *OsGF14C*-OX plants, which may be the principal reason for the reduction of blast resistance. This also indicated the complexity between different stress response pathways and the importance for understanding the crosstalk between different stress pathways. According to the results from this study, *LOX2* may play important roles in coordinating salinity tolerance and blast resistance in rice. However, it need more studies to confirm if *LOX2* is the switch gene and how it regulates the salinity tolerance and blast resistance in rice. It is of great significance to study the regulation mechanisms of *LOX2* underlying different stresses in crops.

## Conclusion

**Figure 9:**
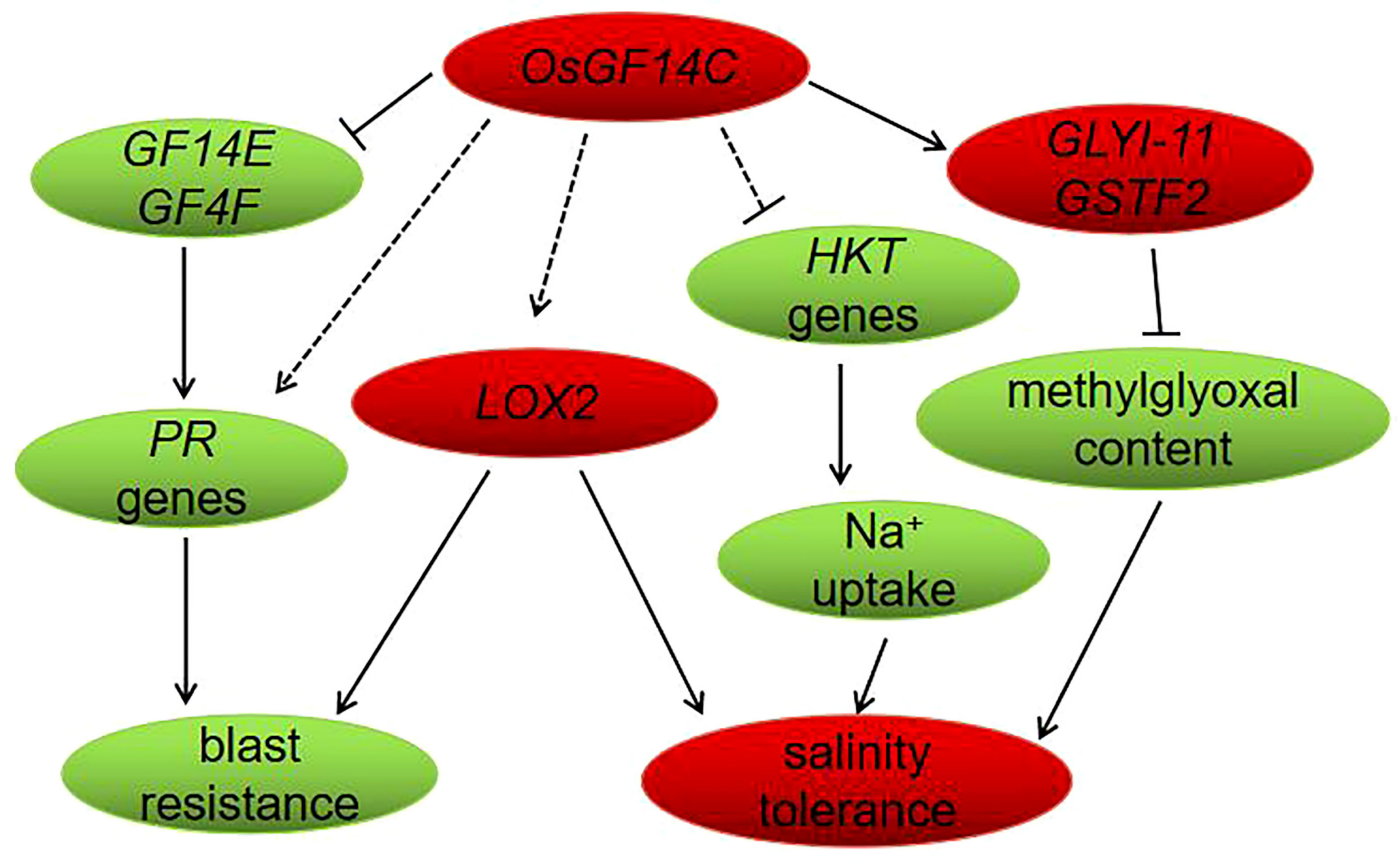
Proposed model of *OsGF14C* in salinity tolerance and blast resistance in rice. When the expression level of *OsGF14C* is up regulated, the expression of *GF14E*, *GF14F*, *PR* genes, and *HKT* genes were repressed, while the expression of *GLYI-11, GSTF2*, and *LOX2* were induced.

In conclusion, in the present study, we have functionally confirmed that overexpression of *OsGF14C* enhances salinity tolerance but reduces blast resistance in rice. The negative role of overexpression of *OsGF14C* plays in blast resistance may be associated with the suppression of *OsGF14E*, *OsGF14F* and *PR* genes. The improvement of salinity tolerance by overexpression of *OsGF14C* may be resulted from reducing the level of methylglyoxal and Na^+^ uptake instead of exclusion or compartmentation. Our results and the previous studies together suggest that the lipoxygenase gene *LOX2* may an important gene which play roles in both abiotic and biotic stresses. Finally, a molecular model of *OsGF14C*-mediated salinity and blast resistance pathways in rice was proposed (**Figure 9**).

## Data availability statement

The original contributions presented in the study are included in the article/**Supplementary Material**. Further inquiries can be directed to the corresponding authors.

## Author contributions

JD conducted the experiments, performed data analysis and wrote the manuscript. XL participated in writing editing and format modification, JY and XZ provided the strain of rice fungus. YM, JC, WY, LiaZ, and JW participated in material development, sample preparation and data analysis. TY, SZ, QL and LinZ provided technical assistance. BL and JZ conceived and designed the experiment, and writing - review & editing. All authors contributed to the article and approved the submitted version.

## Supplementary material

Supplementary Figure 1 Expression analysis of *SOS1*, *NHX1*, and *NHX5* genes in the *GF14C* transgenic plants (OX-9, OX-15) and control plants by quantitative real-time PCR before (0 h) and after (24 h) salinity treatment. Error bars indicate the SD from three biological replicates.
