# Supplementary material for: Overexpression of OsGF14C enhances salinity tolerance but reduces blast resistance in rice
Source: Front Plant Sci. 2023 Feb 10;14:1098855. doi: 10.3389/fpls.2023.1098855 (PMC9950408; doi:10.3389/fpls.2023.1098855)
Supplement: Supplementary file 1 [file Table_1.docx]

| **Supplemental Table 1. Primers used for vector construction and quantitative real time PCR.** | | |
| --- | --- | --- |
|  |  |  |
| Gene | Forward primer (5'-3') | Reverse primer (5'-3') |
| *OsGF14C*-OX | CGCCACCGAAGTAATCCCTT | CCAATAACATGCGGAGCCAT |
| *OsGF14C*-GFP | ATGTCTCGGGAGGAGAATGT | CTGGCCCTCGCAGGCGTCGC |
| *Actin* | CACATTCCAGCAGATGTGGA | GCGATAACAGCTCCTCTTGG |
| *CHT1* | TCCGACTACTGTGTCCAGA | TGTAGGAGATCTGGATGGGT |
| *GF14c* | ACTGCTGCAGAATCTAAGGTG | CCAGAGCAATATCCTGAGCA |
| *GF14E* | ATCCTCAAGCTTCTTGACTC | CTCCTCTCAGCTCCAGTCTT |
| *GF14F* | AAGCTTCTGGATTCCCACCT | AGCAGCTGAGAACACTCTTGTGG |
| *GLYI-11* | GTGAACCTGGTCCTGTCAA | CCACACGAAGCATAACTTGG |
| *GSTF2* | CCAGAGCTGTTGAAGGATG | GGACGGATTAGGCACTGAA |
| *HKT1* | CAACATTTGCACTGTCCAATG | AAAGACTGAGATGCAGGCAAG |
| *HKT1;1* | ATGAGCTCGTCTCAAGGTG | CAGAATGGGTCAAGATTGG |
| *HKT4* | CAGGGTAAGCCAGGTCCAA | AGGATCAGAACCCAGAGTTG |
| *HKT5* | ATCACCTTCGACGAGAAGGA | TGAGCCAACATATTGCGGCA |
| *HKT6* | GTGAGCTTGATCATTCTGC | CCTAGCATTGACCGACTGA |
| *LOX2* | GCATCCCCAACAGCACATC | AATAAAGATTTGGGAGTGACATATTGG |
| *NHX5* | GGCGATTTCGCTTTACAGGAC | TCTGAGCTGTGATGCAACCT |
| *OsNHX1* | TAACAAAGAAGGCACCGAATG | GCTGAGTATGGCCAGATCTTG |
| *OsSOS1* | AAAATGGCAACACATGAGCTC | TAAGTTCAAGTCGGCATTTGG |
| *PR1* | AGAAGCAGTGGTACGACCA | AGGCTGTTGTCGCAGACGA |
| *PR10a* | CCCTGCCGAATACGCCTAA | CTCAAACGCCACGAGAATTTG |
| *PR5* | CTGCAGGGACAGCAGATG | ACTTGGTAGTTGCTGTTTCCC |
| *SUS1* | ACAGGTTCCAGGAACTTGGT | CCATTGGGATTGTTCCAAGG |
| *TUBB3* | CTCTGCAACAATGAGTGGTG | GAGCGAATCCAACCATGAAG |
