# Supplementary material for: Overexpression of OsGF14C enhances salinity tolerance but reduces blast resistance in rice
Source: Front Plant Sci. 2023 Feb 10;14:1098855. doi: 10.3389/fpls.2023.1098855 (PMC9950408; doi:10.3389/fpls.2023.1098855)
Supplement: Supplementary file 2 [file Table_2.docx]

| **Supplemental Table 2. The RNA-seq results of *OsGF14C* client genes in *OsGF14C*-OX plants.** | | | | | | | |
| --- | --- | --- | --- | --- | --- | --- | --- |
| Gene | *OsGF14C*-OX_normalize | CK_normalize | Log_2_FoldChange | pval | Up/Down | Significant | GeneName |
| OS01G0633100 | 51.86027421 | 78.49147867 | -0.597906204 | 1.50E-19 | down | no | *OS01G0633100* |
| OS01G0764000 | 5.770905687 | 1.963355925 | 1.555476019 | 2.03E-07 | up | yes | *GSTF2* |
| OS02G0121300 | 414.6058475 | 429.4841086 | -0.050864287 | 0.057044188 | down | no | *OS02G0121300* |
| OS02G0580300 | 56.1351735 | 140.0293494 | -1.318752308 | 3.34E-113 | down | yes | *GF14E* |
| OS02G0626100 | 3127.755935 | 2393.120513 | 0.386234891 | 4.82E-297 | up | no | *PAL* |
| OS03G0177900 | 111.4459319 | 107.1431376 | 0.056804505 | 0.279564103 | up | no | *REFA3* |
| OS03G0401300 | 32.15218883 | 65.70230363 | -1.031026999 | 4.13E-37 | down | yes | *SUS1* |
| OS03G0401366 | 1.498936542 | 1.051797817 | 0.5110819 | 0.297040037 | up | no | *OS03G0401366* |
| OS03G0710800 | 54.33644965 | 142.9042634 | -1.395056751 | 1.10E-125 | down | yes | *GF14F* |
| OS04G0462500 | 3.907280934 | 3.23088411 | 0.274235986 | 0.327588497 | up | no | *GF14B* |
| OS05G0208000 | 10.79830367 | 12.6067831 | -0.223395492 | 0.148411281 | down | no | *OS05G0208000* |
| OS05G0402700 | 101.3051384 | 107.125981 | -0.080601063 | 0.119209153 | down | no | *OS05G0402700* |
| OS05G0405000 | 281.1111028 | 312.1287453 | -0.151000792 | 8.54E-07 | down | no | *PPDK1* |
| OS06G0644200 | 1.989161203 | 3.357585448 | -0.755263918 | 0.021479275 | down | no | *OS06G0644200* |
| OS06G0671900 | 80.41794548 | 26.57542484 | 1.597424723 | 4.65E-87 | up | yes | *TUBB3* |
| OS08G0191700 | 37.6233072 | 6.941865592 | 2.438231335 | 2.99E-71 | up | yes | *GLYI-11* |
| OS08G0430500 | 316.6503445 | 89.89365342 | 1.81659948 | 0 | up | yes | *GF14C* |
| OS08G0500700 | 291.4121162 | 320.7444362 | -0.138363378 | 4.59E-06 | down | no | *OS08G0500700* |
| OS10G0167300 | 72.69842229 | 53.36120925 | 0.446132694 | 1.44E-10 | up | no | *OS10G0167300* |
| OS11G0186200 | 115.717901 | 69.41865592 | 0.737216723 | 5.26E-37 | up | no | *OS11G0186200* |
| OS11G0210500 | 0.674521444 | 1.121917671 | -0.734030598 | 0.212535154 | down | no | *ADH2* |
| OS11G0455500 | 644.6926067 | 551.4225355 | 0.22545321 | 1.16E-23 | up | no | *OS11G0455500* |
| OS12G0124200 | 105.1503984 | 88.28089677 | 0.252281127 | 6.50E-06 | up | no | *RPS16A* |
