# Supplementary figures and images for: Overexpression of OsGF14C enhances salinity tolerance but reduces blast resistance in rice
Source: Front Plant Sci. 2023 Feb 10;14:1098855. doi: 10.3389/fpls.2023.1098855 (PMC9950408; doi:10.3389/fpls.2023.1098855)

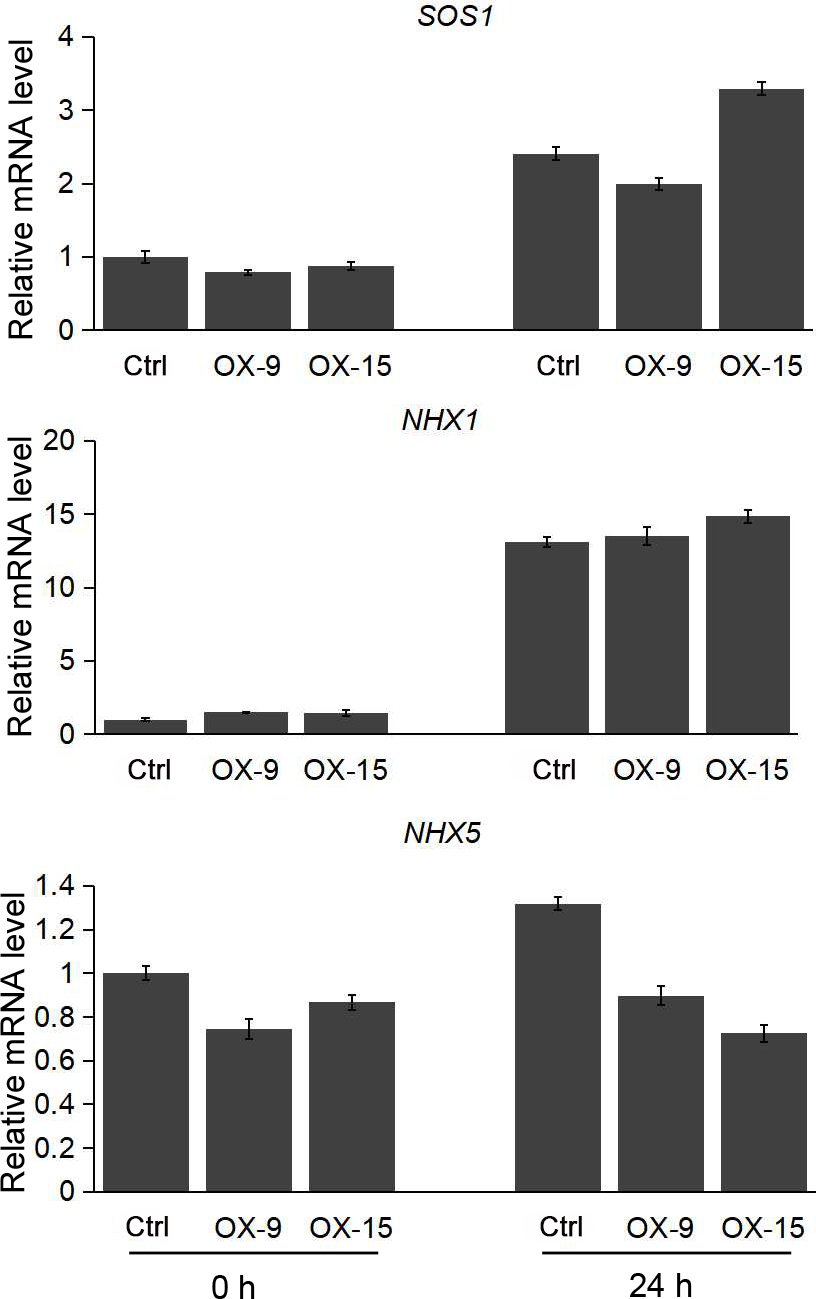

Supplement: Supplementary Figure 1 — Expression analysis of SOS1, NHX1, and NHX5 genes in the GF14C transgenic plants (OX-9, OX-15) and control plants by quantitative real-time PCR before (0 h) and after (24 h) salinity treatment. Error bars indicate the SD from three biological replicates. [file Image_1.tif]
